# Tomorou attenuates progression of rheumatoid arthritis through alteration in ULK-1 independent autophagy pathway in collagen induced arthritis mice model

**DOI:** 10.1038/s41420-019-0222-2

**Published:** 2019-11-07

**Authors:** Arooma Jannat, Peter John, Attya Bhatti, Muhammad Qasim Hayat

**Affiliations:** Atta-ur-Rahman School of Applied Biosciences (ASAB), National University of Science and Technology (NUST), Islamabad, 44000 Pakistan

**Keywords:** Autoimmunity, Pharmacology, Molecular biology, Cell death and immune response, Interleukins

## Abstract

Rheumatoid arthritis (RA) is a multifactorial disease which is complicated by apoptosis resistance. Autophagy is one of the key mechanisms which are involved in the development of resistance to apoptosis as well as to the standard therapies against RA. Aberration in autophagy and apoptosis homeostasis results in the development of oxidative stress thus complicates the pathogenesis of RA. In the given study, tomorou, an indigenous herb of Hunza-Nagar Valley, has been evaluated for its pro-apoptotic, anti-inflammatory, and anti-rheumatic activity. Several major classes of bioactive phytochemicals including steroids, terpenoids, phenols, flavonoids, and essential oils have been detected in the aqueous and ethyl acetate extracts of tomorou through phytochemical analysis. Plant extracts depicted enhanced free radical scavenging activity through di-phenyl-2-picryl hydrazyl hydrate (DPPH) assay and ameliorated the symptoms of arthritis in collagen induced arthritic (CIA) mice model. Moreover, the 6 week extract treatment resulted in the reduction of IL-6 serum levels thus making it an effective anti-inflammatory agent. Upregulation of microtubule-associated proteins light chain 3b (LC3b) and downregulation of UNC51-like kinase 1 (ULK-1) in arthritic mice proposed a ULK-1 independent non-canonical autophagy pathway. Treatment with extracts upregulated the expression of caspase 3 which in turn inhibited the activity of LC3b thus altering the autophagy pathway. However, ULK-1 expression was restored to normal in aqueous extract treated group whereas it was upregulated in ethyl acetate extract treated group. On the other hand, a novel LC3b-independent autophagy pathway was observed in mice treated with ethyl acetate extract due to ULK-1 upregulation. Despite of significantly high IL-6 levels, the arthritic symptoms waned off which suggested the participation of IL-6 in LC3b-independent autophagy pathway in the extract prepared in ethyl acetate. Conclusively, the study established pro-apoptotic, antioxidant, anti-inflammatory and anti-rheumatic activity of tomorou and suggested an intricate autophagy pathway shift.

## Introduction

Rheumatoid arthritis (RA) is an autoimmune disease of obscure etiology that is delineated by hyperplasic synovium, bone erosion, and disrupted joint architecture due to the presence of citrulinated proteins-autoantibody complexes^1,2^. The persistent articular damage and synovial thickness is attributed to the infiltration of inflammatory cells which manifest pannus formation^3^. The abundance of apoptosis resistant fibroblast-like synoviocytes (FLS) at pannus-cartilage junction leads to the development of a cytokine and chemokine milieu in the extracellular matrix (ECM) and contributes to the joint degradation^4–8^. Moreover, cellular proliferation, angiogenesis and altered glucose metabolism causes hypoxia thus generating oxidative stress through reactive oxygen species (ROS) and augment the production of citrulinated proteins^9,10^.

The oxidative stress generated contributes to the mitochondrial dysfunction ultimately disrupting the balance between apoptosis and autophagy^11,12^. Although there are multiple mechanisms to confer apoptosis such as blockade of caspase 3, caspase 7, and caspase 9 activity by the action of inhibitors of apoptosis proteins (IAPs) but enhanced autophagy through increased activity of autophagy related genes (ATGs), microtubule-associated proteins 1A/1B light chain 3 (LC3) and Beclin-1, play vital role in this phenomenon^13–15^. Hyper-activation of autophagy is involved in increased production of cirtulinated proteins, osteoclastogenesis, and survival of lymphocytes in RA synovium^16–18^. In addition, presence of peptidylarginine deiminase (PAD) has been observed in autophagic compartments thus indicating the increased production of citrulinated proteins that complicate RA pathogenesis^19,20^. Resistance to methotrexate treatment has also been attributed to increased autophagy^21^. Moreover, studies show that inhibition of autophagy ameliorate synovial hyperplasia, inflammation, and pannus growth^15,21,22^. This attenuation of synovial inflammation can be either due to reduced hypoxia and initiation of PI3K/AKT pathway mediated apoptosis or culmination of UNC51-like kinase 1 (ULK-1) mediated autophagy initiation complex by the action of mTOR^18,23,24^.

Cytokines are known to alter the microenvironment along with disrupting the ongoing cellular mechanisms and lead to the development of autoimmune conditions^25^. Interleukin 6 (IL-6), the pleiotropic pro-inflammatory cytokine has been known to disrupt the homeostasis of osteoblasts and osteoclasts thereby contributing to the persistent joint damage and hypoxia^26^. Studies also indicate that IL-6 has pivotal role in aggravating autophagy and influencing IL-17 activity through mTOR, janus kinase/signal transducer and activator of transcription (JAK-STAT) and AMPK pathway leading to persistent joint damage^27–29^. Although, biologics including tumor necrosis factor (TNF) inhibitors (TNFi), rituximab (B-cell depletors), CTLA4-Ig (T-cell activation blockers), and tocilizumab (IL-6 inhibitor) have shown promising effect in comparison to standard disease modifying anti-rheumatic drugs (DMARDs) or methotrexate therapy but still there are apprehensions regarding treatment resistance^30,31^. Moreover, despite of high efficacy and effectiveness of tocilizumab there are still concerns pertaining to disease remission and treatment failure^32^. Despite of the skepticism, the use of folk medicine either as a monotherapy or in combination with standard therapy against inflammatory conditions is increasing^33^. The abundance of phytochemicals, in plants, synergistically modulate the immune response by directly influencing the signaling cascades such as *Salix alba* acts as an inhibitor of Nuclear Factor kappa B (NF-κB), cyclooxygenase (COX), and pro-inflammatory cytokines^34^. Recently, plants of family Lamiaceae have shown apoptosis of human breast cancer cells by the activity of caspase 3 and caspase 7^35^. Furthermore, teas and infusions from different plants are being extensively evaluated for their anti-rheumatic, anti-inflammatory, and anti-hypertensive activities^36–40^.

By the virtue of drug resistance in RA, autophagy-apoptosis homeostasis is part aggressive investigations^21^. Despite of being famous for efficacy against inflammatory conditions, the ethnobotany of Hunza-Nagar Valley, Pakistan is yet to be explored. In the given study, we intend to elucidate the efficacy of an indigenous herb, tomorou against rheumatoid arthritis and to delineate the altered autophagic pathways that complicate the disease pathogenesis and prognosis. In order to elucidate that, plant extracts in organic (ethyl acetate) and inorganic (water) solvents were prepared and administered to the collagen induced arthritic (CIA) mice model. The treatment attenuated the arthritic symptoms and delineated the autophagic mechanisms that complicated the disease pathogenesis and prognosis. Thus, the treatment proved to be efficacious and effective against the disease.

## Results

### Discovery and molecular phylogenetic identification of the plant

Tomorou is an indigenous plant of Hunza-Nagar Valley that grows on altitude ≥ 12,000 ft. Traditionally infusions and tea prepared from the plant are used as a local cure for hypertension, obesity, common cold, throat inflammation, and diabetes. Moreover, native people of Hunza-Nagar Valley use it regularly, so there is a possibility that the infusions from the plant had low toxicity.

**Fig. 1:**
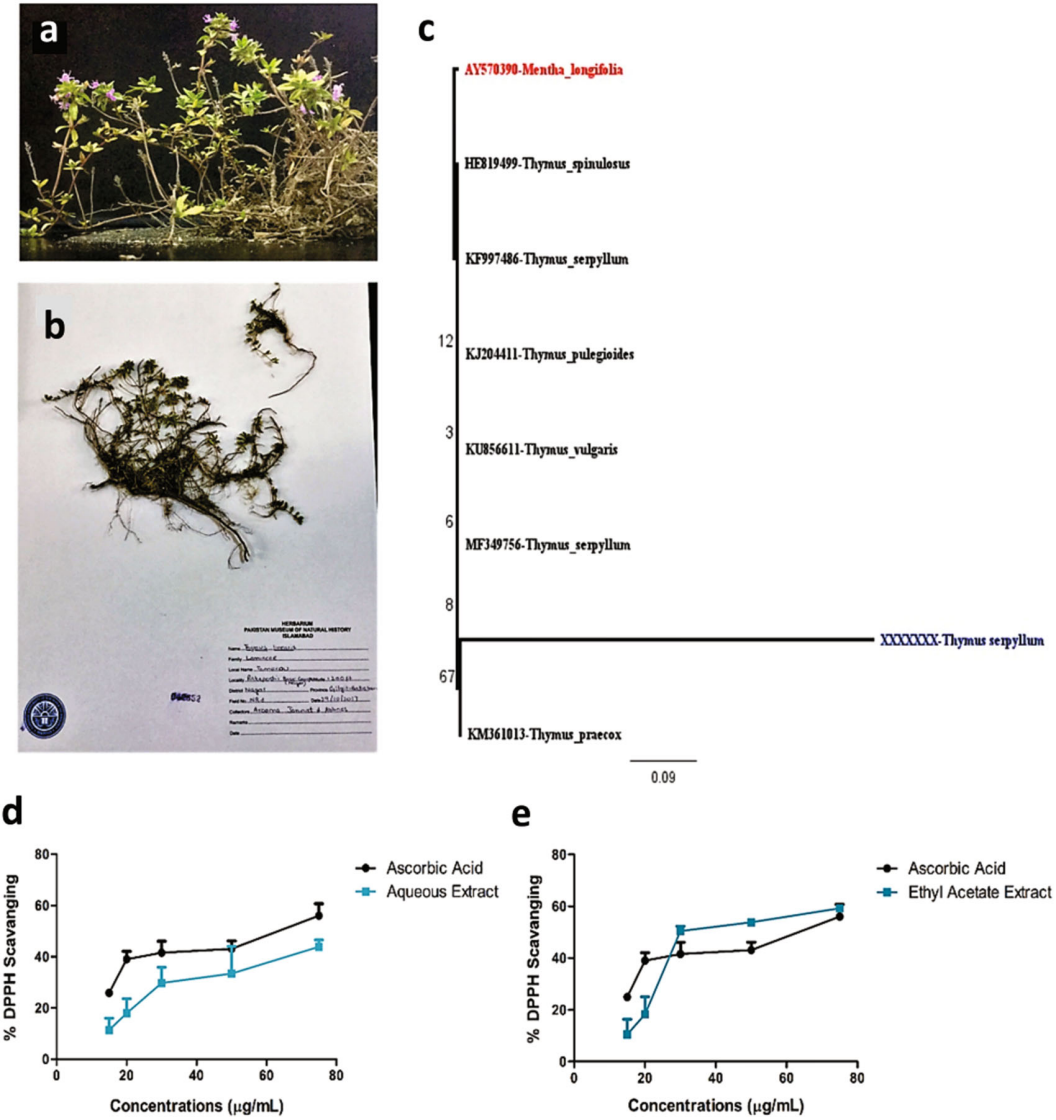
Plant Identification and its antioxidant activity. **a** Plant collected from Rakaposhi base camp. **b** Herbarium submitted to Pakistan Museum of Natural History. **c** Phylogenetic tree generated through Maximum likelihood method with 0.9 base/substitution indicating variation of the plant collected with already reported species. **d** The free radical scavenging activity of aqueous extract of *Thymus serpyllum* increased with increasing concentration of the extract thereby proving it a potent antioxidant. **e** The free radical scavenging activity of the ethyl acetate extract exceeded to that of the standard at initial concentrations but then it was equivalent to the standard as the concentrations increased. **d**, **e** Data were obtained from three independent observations and has been presented as mean ± SD and linear regression was performed

The plant sample was collected from Rakaposhi base camp, Nagar Valley, Pakistan and after morphological analysis, the herbarium sample was submitted to Pakistan Museum of National History under the voucher number 042852 (Fig. 1a, b). In order to classify the plant, *rbcL* gene was amplified, sequenced and analyzed. Although the analysis revealed similarity index of 99% with genus Thymus but the 0.9/base substitution indicated variation in this new variant of *Thymus serpyllum* (Fig. 1c). The results also depict the close relationship of tomorou with *Thymus praecox* and *Thymus serpyllum*. The novel sequence has been submitted to genbank national center for biotechnology information (NCBI) under the accession number MK105914.

### Screening of phytochemicals and free radical scavenging activity of the plant extracts

The plant extracts prepared in, water and ethyl acetate were screened for the presence of phytochemicals before their administration as treatment to CIA mice model. A plethora of phytochemicals including flavonoids, steroids, sterols, terpenoinds, phenols, anthraquinones, alkaloids, glycosides, coumarins, amino acids and etc. were identified in the extracts (Table 1). Due to the presence of different phytochemicals both extracts depicted free radical scavenging activity. The aqueous extract (TS-Aq) and ethyl acetate extract (TS-EA) exhibited a continuous pattern of increased free radical scavenging activity with respect to the increasing concentration (Fig. 1d, e). The pattern of free radical scavenging implicitly established the role of extracts as potential antioxidants.

**Table 1:** Phytochemical analysis of *Thymus serpyllum* extracts

Phytochemical	Aqueous extract	Ethyl acetate extract
Alkaloids	++	++
Phenols	+++	++
Anthraquinones	++	−
Flavonoids	+++	+++
Anthocyanins	−	−
Leucoanthocyanins	−	−
Tannins	+++	++
Phlobatannins	+	−
Coumarins	++	+++
Terpenoids	++	−
Diterpenes	+	+++
Triterpenes	+	+
Steroids	+++	+++
Sterols	+++	++
Saponins	−	+
Resins	++	−
Glycosides	+++	−
Cardiac glycosides	+	++
Protein	+	−
Amino acids	+++	++
Carbohydrates	++	−
Deoxysugar	++	+

Note: The strength of phytochemical has been evaluated in comparison to *Nigella sativa*

**−** not present, + weakly present, ++ moderately present, +++ strongly present

### Extracts attenuate the arthritic symptoms and extra-articular manifestation

**Fig. 2:**
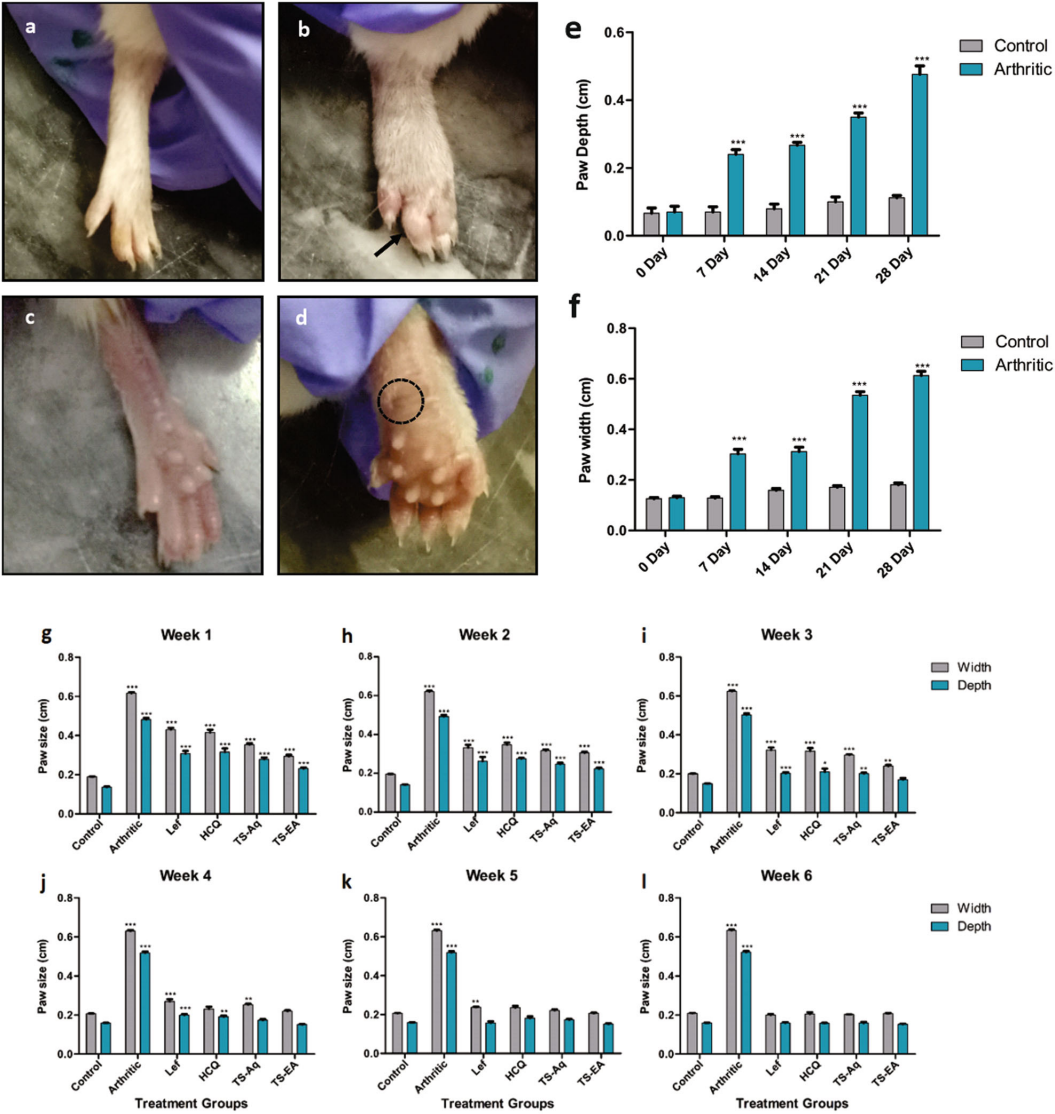
Arthritis induction and treatment prognosis. **a** Front view of Normal paw indicating no swelling (arthritic score 0). **b** Arthritic mice paw showing signs of ankylosis, swelling and joint deformation (arthritic score 4). **c** Back view of normal paw showing no signs of rheumatic nodules. **d** Arthritic mice paw showing rheumatic nodule formation, swelling and severe abscission denoted by a dotted circle. **e** From day 7–28, the paw edema increased significantly in arthritic mice in comparison to control mice (*p* = < 0.0001). **f** The paw width of arthritic mice significantly (*p* = < 0.0001) deviated from those of normal mice. **g** On day 30, treatment groups were administered oral dose of standard drugs and extracts and minimal difference was observed in paw size of treated groups as compared with arthritic group at the end of week 1. **h** During week 2 paw sizes started to reduce but no significant difference was observed in treated groups as compared with arthritic group. **i** During week 3 of treatment ethyl acetate extract started its effect by reducing the paw size that is nearly equal to the paw size of control group. **j** During week 4 aqueous extract and HCQ treated mice started to reduced paw size to normal whereas ethyl acetate extract further reduced paw size to normal. **k** During week 5 all the treated groups began to reduce paw edema. The paw size of all treatment groups became nearly equal to the paw size of the control group. **l** On week 6, the paw measurement revealed that paw edema diminished in all treatment groups. **a**–**d** Dotted circle indicate abscess whereas arrow denotes ankylosis. **e**–**l** Data were obtained from three independent observations and has been presented as mean ± SEM and student’s *t*-test was performed. Data were obtained from three independent observations and has been presented as mean ± SEM and student’s *t*-test was performed to calculate statistically significant difference between control group and other experimental groups. *p*-value < 0.05 was considered to be statistically significant. *p < 0.05; **p < 0.01; ***p < 0.001; ****p < 0.0001. The dose administered is; aqueous extract of *Thymus serpyllum* (TS-Aq) and ethyl acetate extract of *Thymus serpyllum* (TS-EA) is 660 mg/kg, Leflunomide (Lef) is 10 mg/kg and Hydroxychloroquine (HCQ) is 25 mg/kg

In order to investigate the effect of different extracts on arthritis induction and progression, arthritis was induced using protocol explained by William et al.^41^. The arthritic mice were administered extracts and standard drugs orally. During arthritis induction the paw depth and width continue to increase significantly versus control, thus confirming arthritis generation (Fig. 2e, f). On day 28, joint modification, extensive swelling, ankylosis, arthritic nodule formation and severe abscess formation was observed in the paw of arthritic mice (Fig. 2c, d) whereas the control group had normal paw morphology and physiology (Fig. 2a, b). The arthritic mice were administered with extract and standard drug treatment on day 30. After 3 weeks of treatment with extracts the arthritic symptoms started to ameliorate. Although there was no significant decrease in the paw depth and width in comparison to arthritic mice but joint inflammation began to reduce after a week (Fig. 2g, h). The extract prepared in ethyl acetate significantly reduced the paw depth during third week of treatment whereas the extract prepared in water began to significantly reduce the inflammation in fourth week of treatment (Fig. 2i, j). However, the standard drugs produced their effect during fifth week of the treatment (Fig. 2k). In sixth week of treatment all the groups except the arthritic group depicted attenuation of swelling and inflammation (Fig. 2l). Joint inflammation and swelling reduced in the treatment groups indicating the treatment efficacy. Results indicate that extracts were more efficacious than the standard drugs used during the study.

**Fig. 3:**
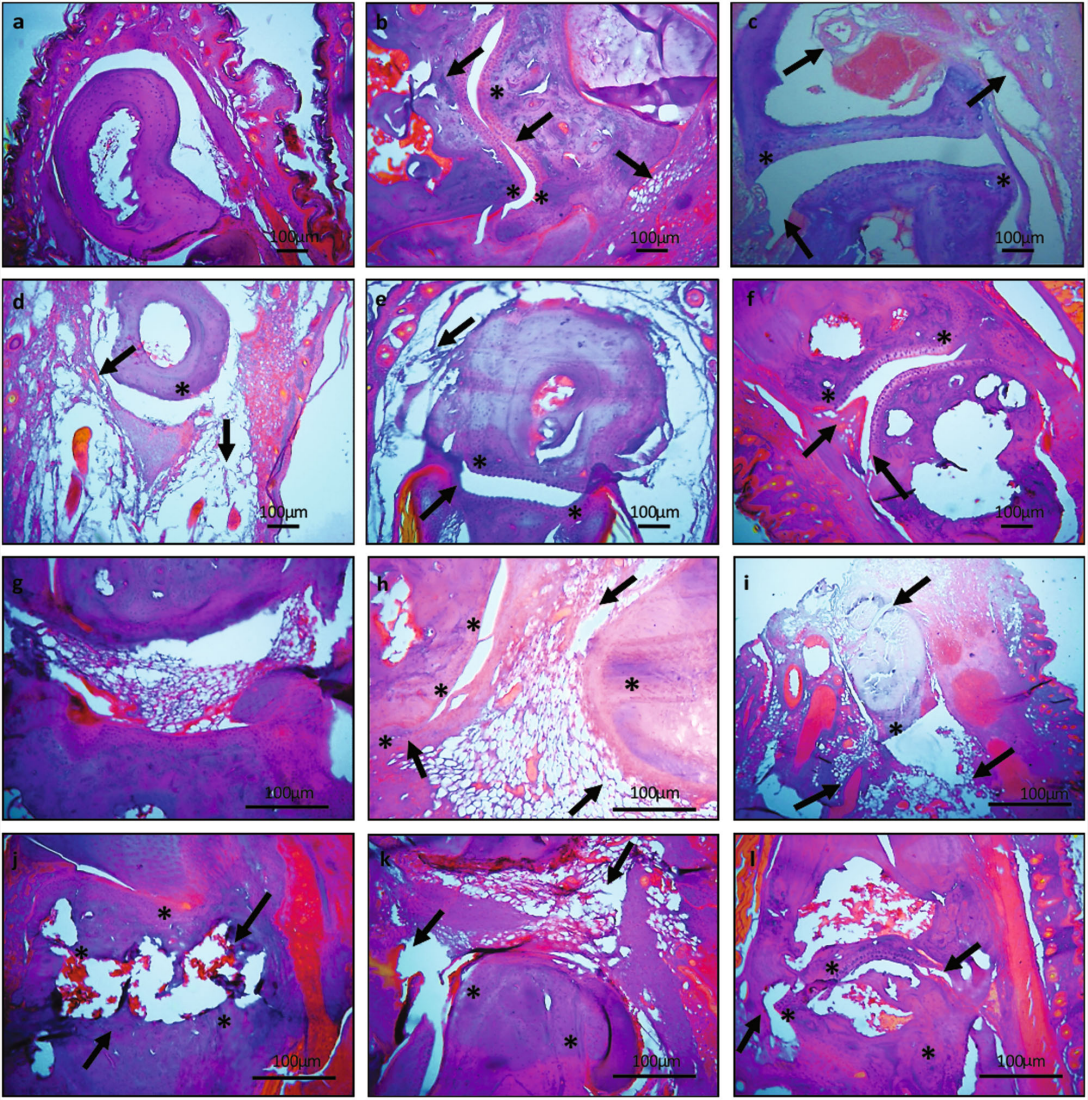
H&E staining of ankle and tarsal joints. **a** Normal mice tarsal joint revealed intact cartilage and no signs of bone erosion. **b** Arthritic tarsal joint exhibited joint modification, continuous bone erosion and cellular infiltration. **c** Hydroxychloroquine treated mice depicted diminished cellular infiltration and pannus formation along with progressive bone erosion of tarsal joint. **d** The tarsal joint of leflunomide treated group had modification in joint architecture but reduced cellular infiltration. **e** There was less cellular infiltration and diminished joint modifications after aqueous extract administration. **f** The ethyl acetate extract treated mice had preserved joint structure along with controlled cellular infiltration. **g** Normal ankle joint. **h** Arthritic ankle joint exhibited cellular infiltration joint modification and continuous cartilage degradation. **i** Hydroxychloroquine treated mice depicted progressive cartilage degradation. **j** The ankle joint of leflunomide treated group had joint modification along with cartilage distortion. **k** The cartilage and joint architecture was preserved along with less cellular infiltration in ankle joint of aqueous extract treated mice. **l** However, the ethyl acetate extract treated mice had preserved joint architecture and less cartilage distortion but still there were morphological changes. Arrows indicate joint modification, pannus formation, cartilage, and bone erosion whereas * indicate cellular infiltration. Original magnifications ×20 and ×40. Scale bars are shown below

Next we evaluated the joint morphology through histopathological analysis. The histological sections of arthritic hind and tarsal joints depicted joint deformity, cartilage degradation, pannus formation, cellular infiltration indicating persistent joint damage (Fig. 3b, h). Whereas the normal paws had adequate joint space, intact boundaries and cartilage with no indication of cellular infiltration (Fig. 3a, g). The extract treated groups had preservation of joint architecture whereas the standard drug treated groups depicted significant bone loss. Although joint swelling was reduced but the joint damage remained persistent in hydroxychloroquine (HCQ) treated groups (Fig. 3c, i). However, treatment with leflunomide (Lef) resulted in diminished cellular infiltration but joint degradation remained constant (Fig. 3d, j). Aqueous extract ameliorated joint damage along with preservation of joint architecture (Fig. 3e, k) whereas the ethyl acetate extract attenuated hyperplasia and reduced cellular infiltration but there were still modifications in the joint structure (Fig. 3f, l). We observed reduction in cellular infiltration, hyperplasia, pannus growth, and inflammation in the extract treated groups whereas there were joint modifications in standard drug treated groups.

**Fig. 4:**
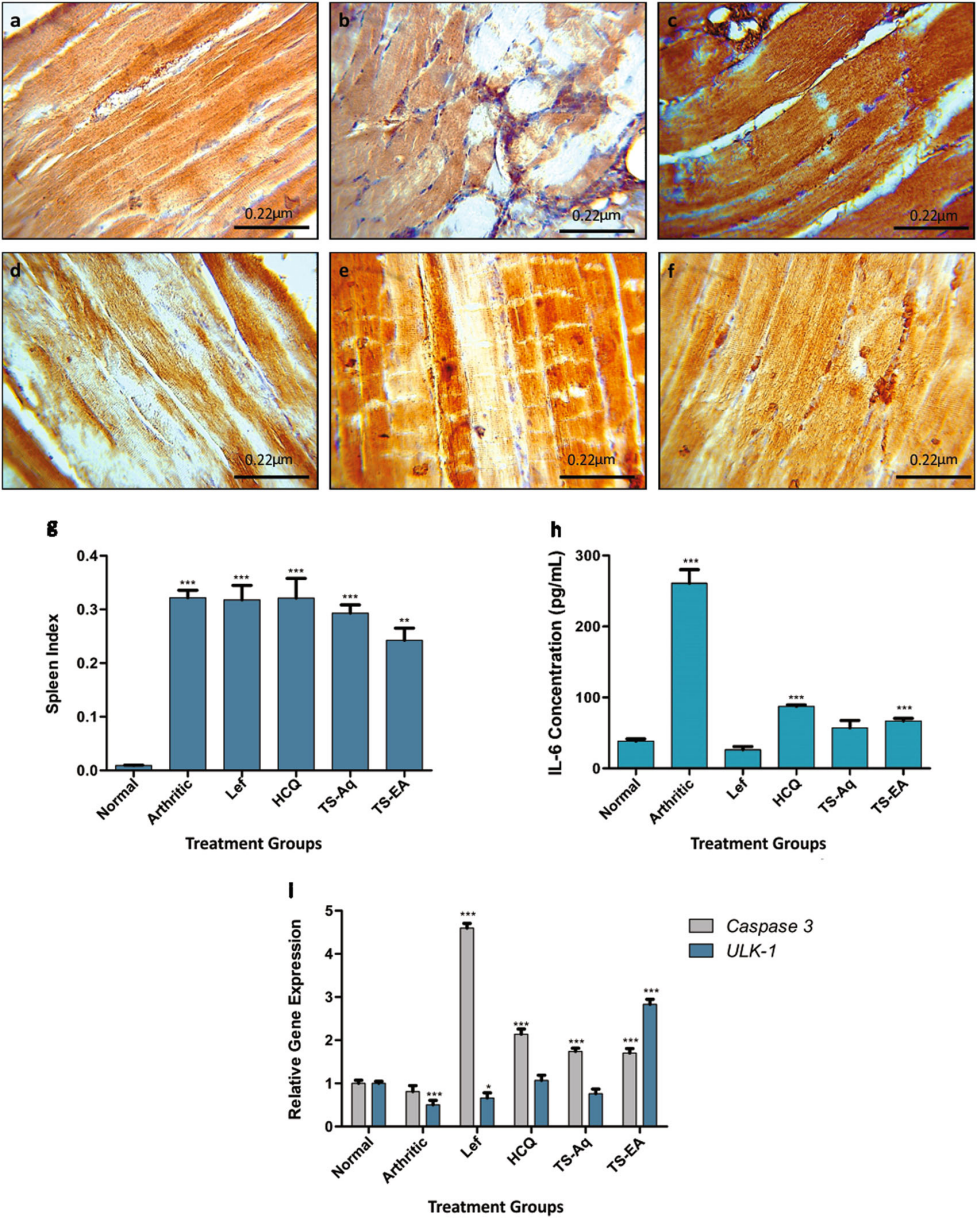
Biochemical, physiological and molecular analysis to establish anti-inflammatory and regulatory role of the treatment. **a** Normal levels of LC3b observed in IHC. **b** Whereas the arthritic mice had significantly elevated LC3b expression indicative of autophagy. **c** The hydroxychloroquine treated mice depicted inhibition of autophagy as LC3b was diminished. **d** Leflunomide interfered with autophagy through inhibition of LC3b. **e** The aqueous extract also indicated low LC3b levels thus suggesting inhibition of autophagy. **f** However, the ethyl acetate extract treated mice depicted inhibition of LC3b but autophagy was observed. **g** The spleen indexing indicated reversal of splenomegaly after ethyl acetate treatment whereas other groups had aggressive splenomegaly. **h** The extract treatment significantly reduced the IL-6 levels to the normal whereas the standard drugs either dropped it below the normal or the levels were higher enough to be detrimental. **i** The expression analysis revealed the upregulation of Caspase 3 and ULK-1 after the administration of ethyl acetate extract indicating restoration of autophagy-apoptosis homeostasis whereas the aqueous extract restored the ULK-1 expression to normal but instigated apoptosis through Caspase 3 upregulation. The hydroxychloroquine and leflunomide halted autophagy and induced apoptosis. However, the arthritic group indicated downregulation of ULK-1 expression with halted apoptosis. **a**–**f** Original magnifications ×40. Scale bars are shown below. **g**–**i** Data were obtained from three independent observations and has been presented as mean ± SEM and student’s *t*-test and ANOVA were performed to calculate statistical significant difference between control and other experimental groups. *p*-value < 0.05 was considered to be statistically significant. *p < 0.05; **p < 0.01; ***p < 0.001; ****p < 0.0001

We further assessed spleen enlargement in order to confirm extra-articular involvement. Results indicated severe splenomegaly in arthritic mice. However, after the treatment the spleen enlargement remained as a constant feature in standard drugs treatment groups (Fig. 4g). Whereas treatment with aqueous and ethyl acetate extracts depicted less aggressive splenomegaly, thus implying that extracts are a better approach for treatment.

### Extracts as anti-inflammatory agents

IL-6 is known to aggravate arthritic symptoms as it disrupts the homeostasis of osteoblast and osteoclast leading to persistent joint damage^26^. In order to validate the anti-inflammatory activity of extracts IL-6 levels were measured. The aqueous extract treated group, had normal IL-6 levels whereas the ethyl acetate extract treatment had significantly higher IL-6 levels. The standard drug treatment, leflunomide, decreased the IL-6 levels below normal whereas hydroxchloroquine treated groups had elevated IL-6 levels (Fig. 4h). Our results established anti-inflammatory role of the extracts. Moreover, these altered IL-6 serum levels indicated remodeling of downstream cellular pathways.

### Regulation of autophagy and apoptosis

**Fig. 5:**
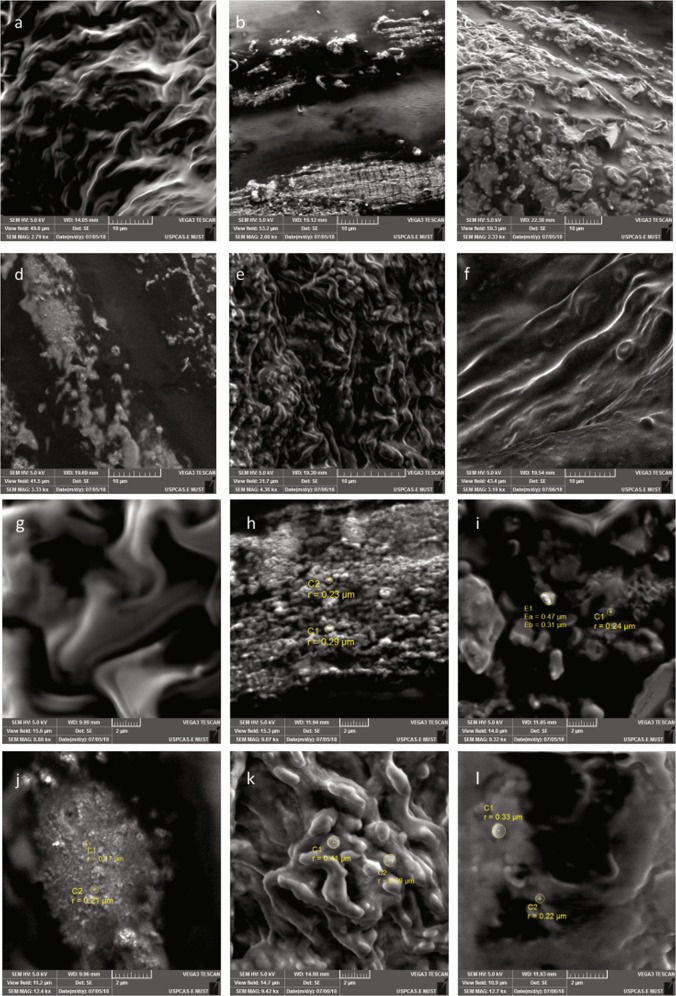
Scanning electron micrographs showing cellular morphology of skeletal muscles along with signs of apoptosis and autophagy. **a** Normal skeletal muscles had no signs of cellular degeneration. **b** Whereas the arthritic muscles had signs of aggressive muscular degeneration. **c** Hydroxychloroquine treated mice explicitly demonstrated signs of apoptosis in skeletal muscles. **d** The leflunomide treated group had signs of apoptosis but there were few indications of cell engulfing. **e** There are evident signs of apoptosis in aqueous extract treated mice. **f** The ethyl acetate extract treated mice had morphology near to normal with evident signs of restored balance of autophagy and apoptosis. **g** Normal skeletal muscles indicated no signs of aggressive apoptosis and autophagy. **h** Arthritic mice depicted signs of aggressive autophagy and engulfing of cellular debris due to muscular disintegration. **i** Hydroxychloroquine treated mice depicted progressive apoptosis as inferred from blebs observed in the micrograph. **j** The leflunomide treated group indicated signs of apoptotic bleb formation but the autophagy seemed diminished. **k** The skeletal muscles of aqueous extract treated mice had explicit signs of apoptosis and clearance of the extracellular matrix through cell engulfing. **l** However, the ethyl acetate treated mice exhibited signs of apoptosis with clearance through autophagy. Scale bars are provided below

To evaluate the modulation in autophagy-apoptosis homeostasis expression of ULK-1, caspase 3 and LC3b was evaluated. Despite of high LC3b expression observed in the skeletal muscles the ULK-1 level was significantly downregulated in arthritic mice thus indicating a pathway shift (Fig. 4b, i). The diminished caspase 3 activity in arthritic mice confirmed increased autophagy along with muscular degeneration in comparison to normal mice (Fig. 5a, b, g, h). Results demonstrate a ULK-1 independent autophagy initiation complex formation in arthritic mice. However, treatment with aqueous extract restored autophagy to normal by acting directly on caspase 3 that inhibited the expression of LC3b (Fig. 4e, i). The ethyl acetate extract treatment upregulated the expression of caspase 3 that inhibited LC3b expression yet there were signs of autophagy as ULK-1 expression was upregulated (Fig. 4f, i). The standard drug, leflunomide diminished autophagy by downregulation of LC3b and ULK-1 and upregulation of caspase 3 (Fig. 4d, i). Whereas hydroxycholorquine treatment restored ULK-1 and LC3b expression to normal but upregulated the expression of caspase 3 (Fig. 4c, i). To further validate the results skeletal muscle dystrophy was observed through scanning electron microscopy (SEM). Despite of diminished skeletal muscle degeneration there were signs of apoptosis in aqueous extract treated group (Fig. 5e, k) but the ethyl acetate extract treated groups had normal muscular morphology (Fig. 5f, l). However, leflunomide treated mice depicted skeletal tissue myopathy (Fig. 5d, j) but there were signs of increased apoptosis in skeletal muscles in hydroxycholorquine treated mice (Fig. 5c, i). Taken together results demonstrate a non-canonical ULK-1 independent autophagy pathway being followed after arthritis induction that is probably responsible for apoptosis resistance. The extract treatment resulted in alteration of this pathway as treatment with ethyl acetate extract resulted in autophagy independent of LC3b whereas the aqueous extract treatment restored the classical canonical autophagy pathway. Thus, implying that alteration in autophagy pathways may result in reduction of arthritic symptoms.

## Discussion

RA is a multifactorial disorder with mysterious pathophysiology that is classified through bone erosion and modifications in joint architecture^1,2^. Due to increased cellular infiltration and production of rheumatoid factor (RF) and anti-citrulinated peptide antibody (ACPA) against the glycosylated proteins induce hypoxia, bone deformation, and pro-inflammatory cytokine production^42–45^. Despite being effective the standard treatments still face the challenge of resistance that is developed in some patients due to autophagy^15^. Herein, we delineate the altered autophagic pathway being followed during the disease progression along with pro-apoptotic, antioxidant, anti-inflammatory, and anti-rheumatic activity of plant extracts. Results signify that treatment with extracts altered the expression of ULK-1, caspase 3, LC3b, and IL-6 which is generally attributed to the presence of high phytochemical content.

Oxidative stress induces alteration in cell signaling network and complicates the pathogenesis of RA^9,21,46^. Increased demand of energy compels a metabolic shift in pathways in RA and causes the accumulation of lactate levels that leads to mitochondrial dysfunction which results in pannus formation, abnormal angiogenesis and cellular infiltration^47–49^. Our study demonstrated reduction in the inflammatory process, bone erosion, and amelioration of arthritic symptoms after administration of extracts. The high antioxidant activity of the extracts is culpable for reduction in the arthritic symptoms and restoration of the normal physiology. Studies have demonstrated that the extracts prepared using aerial parts of *Thymus serpyllum* have promising anti-inflammatory activity^37^. However, the standard drugs treated groups had joint modifications although inflammation and cellular infiltration was halted but bone erosion and joint deformation remained continuous along with diminished IL-6 levels.

The extracts prepared, depicted a plethora of phytochemicals that are produced in the plant probably due to the different types of stresses the plant faces. The presence phytochemicals in the extracts depicted anti-rheumatic activity as symptoms of arthritis assuaged after extract treatment. This was probably due the high antioxidant activity of the phytochemicals present in the extract. Plants of genus Thymus possess essential oils that possess antioxidant, antimicrobial, antitumor, and anti-inflammatory^38^. The presence of these phytochemicals poses antioxidant activity and probably is responsible for the alleviation of classic arthritic symptoms. The cellular infiltration observed during histopathological analysis of arthritic mice suggested increased hypoxia that induced increased IL-6 levels along with disrupted autophagy-apoptosis homeostasis. Despite of ULK-1 downregulation, autophagy was persistent as increased LC3b levels and cell engulfing were evident thus indicating a ULK-1 independent mechanism of autophagy. Studies explicitly demonstrated that cellular infiltration pose hypoxic stress thereby diminishing glucose levels and elevating lactate levels. This increase in lactate levels initiates a non-canonical autophagic pathway^50^. The extracts with antioxidant activity regulated the cellular processes and lead to better treatment of the disease. IL-6 levels dwindled after extract and standard drug administration. The phytochemicals milieu present in the plants act synergistically and produce effect against the oxidative stress generated during disease condition^51^. Recently, canonical to non-canonical autophagy pathway shift has been observed under certain circumstances that is either Beclin-1 independent or ULK-1 independent^50,52,53^. The ULK-1 independent non-canonical autophagy pathway was revealed after administration of plant extracts. However, the mice treated with ethyl acetate extract of *Thymus serpyllum* indicated a pathway shift from ULK-1 independent non-canonical pathway to canonical pathway/classical pathway. However, the autophagic pathway was independent of LC3b. Studies have demonstrated that LC3 lipidation is not required for autophagosome formation, this may result due to blockade of LC3 by the altered pattern of cytokines^54,55^. This pathway shift suggested that autophagy-apoptosis homeostasis was being restored after the extract administration. The antioxidant activity of the extract reduced the hypoxic stress thereby decreasing IL-6 activity, bone erosion, cellular infiltration, alteration of apoptotic, and autophagic pathways.

Increased autophagy contributes to the increased drug resistance in RA patients^21^. Our study provides insight to the ULK-1 independent non-canonical autophagic pathway that complicates the pathophysiology of RA. Administration of extracts altered the autophagic pathway in a way that LC3 independent autophagy was triggered along with diminished arthritic symptoms. Furthermore, the study highlights the anti-inflammatory and antioxidant role of the novel *Thymus serpyllum* variant. The anti-inflammatory property of the plant extracts may be involved in the alteration of autophagy and apoptosis homeostasis. This phenomenon still remains unclear and requires further exploration. Moreover, the amelioration of arthritic symptoms after induction of LC3b-independent autophagy pathway highlights the potential role of LC3 in RA prognosis and may be explored for its therapeutic potential.

## Materials and methods

### Plant collection and identification

Tomoru was collected from Rakaposhi base camp, Hunza-Nagar valley, Gilgit-Baltistan, Pakistan. Herbarium was prepared from whole plant with inflorescence and submitted to Pakistan Museum of Natural History. Molecular phylogenetic analysis was conducted for plant identification. Plant Nucleic acid isolation was done through Doyle and Doyle, with some modifications using leaves as sample^56^. Ribulose bisphospahte carboxylase Large region (*rbcL*) was amplified as conserved region using primer set previously described in Sun et al.^57^ and was sequenced commercially and analyzed to plot a phylogenetic tree.

### Extract preparation and biochemical analysis

Fine ground aerial plant parts were dissolved in solvents water and ethyl acetate (Merck) by 1:10 and kept in dark for 4 weeks with frequent shaking. Extract was filtered and condensed through rotary evaporation. Biochemical tests to qualitatively assess phytochemicals present in the extracts as previously described in Evens and Trease^58^ and Harborne^59^. The strength of phytochemical presence was determined through comparison with *Nigella sativa* extracts. *Nigella sativa* is chosen as a reference because it has been reported to be rich in phytochemicals^60,61^. Di-Phenyl-2-Picryl Hydrazyl Hydrate (DPPH) Assay was conducted by adopting protocol explained by Sanganna et al.^62^ with some modifications.

### Animal procurement, model establishment, and treatment

The experimental procedures and protocols followed were approved by Institutional Review Board at ASAB (IRB-ASAB). 8–12-week-old female *BALB/c* mice, weighing 30–35 g, were housed at Animal Laboratory ASAB, NUST with regulated 16:8 sunlight to darkness. All procedures carried out during the course of study were in accordance with guidelines provided by National Institute of Health (NIH). Collagen Induced Arthritis (CIA) was generated using immunization mix, prepared by dissolving bovine type II collagen (Thermofisher scientific) in 0.1 M acetic acid. Complete Freund’s adjuvant (CFA) (Santa Cruz, Biotechnology) and type II collagen were mixed in 1:1 and vortexed, to this mixture BSA (Bovine Serum Albumin) was added. Mice were injected with the immunization mix in sub-dermal region of the tail on day 0, 7, and 14. On day 21 and 28, a booster dose of CFA was administered in hind paw^41^. From the primary day of immunization, day 0, hind paws were measured using vernier caliper (GmBH) and upon assessing inflammation grade of arthritis was determined. Only those mice that developed 3 or 4 degree arthritis were proceeded for further analysis.

### Administration of *Thymus serpyllum* as treatment

Mice were divided into six groups, 10 mice/group. 660, 10, and 25 mg/kg of aqueous extract, ethyl acetate extract, Leflunomide (Sigma-Aldrich), and Hydroxychloroquine (Sigma-Aldrich) were administered orally to mice through feed, respectively, for 6 weeks. After 6 weeks mice were euthanized, blood and organs were collected. Leflunomide and Hydroxychloroquine were used as standard drugs as both are used to treat RA^63^.

### Histopathology

Histopathological analysis was done in order to evaluate the modifications in the joint architecture. Prior to decalcification for 48–72 h in 5% nitric acid (Merck) resected hind paws were kept in 10% Formalin. Decalcified paws were immersed in different concentrations of ethanol, isopropanol: xylene and pure xylene for 2 h each. After which samples were embedded in paraffin and 5 μm sections were produced using microtome. Slides were sealed with mountant and observed under microscope after staining with hematoxylin (Merck) and eosin (Merck) at ×40.

### Spleen indexing

Spleen index was calculated through measurement of length, width and height of spleen as described in Huang et al.^64^ to evaluate the extent of splenomegaly imposed during diseased condition and treatment.

### Immunohistochemistry

LC3b was taken as a marker for autophagy. Resected thigh muscles were coated with paraffin and 4–6 μm sections were diced. Later samples were deparaffinised and rehydrated for coating with selected antibodies. BSA was used as a blocking agent and LC3 (Abcam) antibody was poured as primary antibody and incubated. After incubation with alkaline phosphatase (Abcam), the secondary antibody, substrate was provided and then counter staining with Mayer’s hematoxylin (Merck) was done and slides were observed under optical microscope at magnification of ×40.

### Real-time polymerase chain reaction

RNA was isolated from blood using TRIzol method^65^. TRIzol (Thermofisher scientific), glacial acetic acid (Sigma-Aldrich), and chloroform (Sigma-Aldrich) were added and centrifuged. RNA was precipitated through isopropanol (Merck) and after elution in DEPC-treated (Sigma-Aldrich) water it was stored at −80 °C. RNA was quantified on NanoDrop (Thermofisher Scientific) and cDNA was synthesized using Oligo dT. ULK-1, Caspase 3, and GAPDH, as housekeeping gene, were quantified using primers reported in Lenhare et al.^66^, Lui et al.^67^, and Ren et al.^68^ in order to monitor alterations in the expression of autophagy and apoptotic markers. Real-time PCR was performed on 7300 Real-Time PCR System (Applied Biosystems). Expression analysis was performed by using ∆∆Ct method explained by Livak^69^.

### Enzyme linked immunosorbant assay (ELISA)

IL-6 was quantified by using ELISA kit (Elabsciences) and by following manufacturer’s instructions to evaluate the anti-inflammatory activity of the extracts.

### Scanning electron microscopy (SEM)

Skeletal muscles were gross sectioned dried and were coated with palladium-gold for scanning electron microscopy (TESCAN Vega3, UK). Coated samples were subjected to electron microscopy at 5 KV and images were taken at various magnifications to observed cell morphology.

### Statistical analysis

GraphPad Prism version 5 for windows (USA) was used to perform statistical analysis. Student *t* test and two-way ANOVA were performed to obtain individual *p* values and assess the association between variables, respectively. Phylogenetic tree was plotted through Maximum Likelihood Method using Geneious © 2005–2019 (Biomatters, New Zealand).

## Supplementary information

Author contribution form
