# Supplementary material for: Tomorou attenuates progression of rheumatoid arthritis through alteration in ULK-1 independent autophagy pathway in collagen induced arthritis mice model
Source: Cell Death Discov. 2019 Nov 7;5:142. doi: 10.1038/s41420-019-0222-2 (PMC6838101; doi:10.1038/s41420-019-0222-2)
Supplement: Supplementary file 1 — Author contribution form [file 41420_2019_222_MOESM1_ESM.pdf]

DECLARATION OF CONTRIBUTIONS TO ARTICLE

ADMC

Manuscript Number: CDDIS-19-0439

Journal Name: *Cell Death & Disease* (the 'Journal')

Proposed Title of the Contribution:

Tomorou Attenuates Progression of Rheumatoid Arthritis through Alteration in ULK-1 Independent Autophagy Pathway in Collagen Induced Arthritis Mice Model (the 'Contribution)

Author(s):

Arooma Jannat, Peter John, Attya Bhatti, Muhammad Qasim Hayat (the 'Authors')

For all *CDDis* articles, each person named as an author in the published version must be able to show he or she has contributed substantially to the article.

Authorship credit should be based on 1) substantial contributions to conception and design, acquisition of data, or analysis and interpretation of data; 2) drafting the article or revising it critically for important intellectual content; and 3) final approval of the version to be published. Authors should meet conditions 1, 2 and 3.

Any person who cannot be shown to have made a substantial contribution to the article cannot be listed as an author in the final version. The name of any person who is deemed to have made a minor contribution can, however, appear in the Acknowledgments section of the article.

Please complete the table below to indicate the contributions of all named authors to the manuscript.

Author Full Name:	Specification of Contribution to the Manuscript:
Arooma Jannat	AJ conceived the idea, conducted plant sampling, experimentation, data analysis and drafted the article along with figures preparation. AJ approved the final version of
Dr. Peter John	PJ supervised the study and provided intellectual content for study design. PJ critically revised the article for errors. PJ approved the
Dr. Attya Bhatti	AB revised the article and provided insight to data analysis and approved the manuscript for publication.
Dr. Muhammad Qasim	MQH revised the manuscript and provided guidance in phylogenetic analysis and interpretation. MQH approved the manuscript for final publication.

Please complete the table below to indicate the contributions of all named authors to the figures.

Figure 1:
AJ and MQH did the phylogenetic analysis and provided guidance in preparation of figure.

Figure 2:
AJ prepared the figure, PJ and AB provided guidance for the improvement.

Figure 3:
AJ prepared the figure. PJ and AB revised the figure for publication.

Figure 4:
AJ prepared the figure. PJ and AB revised the figure for publication.

Figure 5:
AJ prepared the figure. PJ and AB revised the figure for publication.

Figure 6:

Signed for and on behalf of the Author(s):	Print Name:	Date:
	Peter John	12/02/2019
